# Genome-wide association studies identify new candidate genes and tissues underlying resistance to a natural toxin in drosophilids

**DOI:** 10.1093/g3journal/jkag032

**Published:** 2026-02-16

**Authors:** Michele Marconcini, Caroline Fragnière, Ambra Masuzzo, Richard Benton

**Affiliations:** Center for Integrative Genomics, Faculty of Biology and Medicine, University of Lausanne, Lausanne, Vaud, CH-1015, Switzerland; Center for Integrative Genomics, Faculty of Biology and Medicine, University of Lausanne, Lausanne, Vaud, CH-1015, Switzerland; Center for Integrative Genomics, Faculty of Biology and Medicine, University of Lausanne, Lausanne, Vaud, CH-1015, Switzerland; Center for Integrative Genomics, Faculty of Biology and Medicine, University of Lausanne, Lausanne, Vaud, CH-1015, Switzerland

**Keywords:** octanoic acid resistance, *Drosophila*, host–plant specialization, multigenic toxin resistance, genome-wide association, adaptive evolution, chemical ecology

## Abstract

Many insects can rapidly evolve resistance to artificial insecticides through changes in toxin target proteins. Over longer timescales, insects also evolve resistance to naturally occurring toxins to exploit new ecological niches, but the underlying mechanisms often remain poorly understood. A classic example is *Drosophila sechellia*, an extreme specialist for the ripe noni fruit of *Morinda citrifolia*. Noni is toxic for other insects—including *D. sechellia*'s close relatives *Drosophila simulans* and *Drosophila melanogaster—*due to this fruit's high content of octanoic acid (OA). However, the mechanistic bases of OA susceptibility and resistance across species remain unclear. Here, we first show that the species-specific tolerance of OA is independent of these drosophilids’ distinct microbiomes. Screening large, genetically diverse panels of *D. melanogaster* and *D. simulans* strains revealed broad variation in OA resistance, with some lines surviving as well as *D. sechellia*. Resistance to OA does not correlate with resistance of these lines to other insecticides, implying a distinct toxicity mode of action. Genome-wide association and transcriptome-to-phenotype analyses identified multiple genes linked to OA resistance, with diverse expression patterns and functions, including those involved in epithelial septate junction formation and lipid transport. Loss-of-function analysis in *D. melanogaster* confirmed that at least 2 of these—Bez, a CD36-family fatty acid transporter, and CG13003, a putative extracellular matrix component—positively contribute to OA resistance. Integration of our findings with those from previous complementary genetic approaches supports a model in which OA has no singular target, and that resistance is defined by multigenic and multitissue defense mechanisms.

## Introduction

Animals are exposed to a myriad of volatile and nonvolatile chemicals. Some are exploited as beneficial signals, for example, when locating food (Roura and Foster 2018; Ruedenauer et al. 2023) or conspecifics (Wyatt 2014). But many chemicals are toxic, such as heavy metals or those produced by plants as defense against herbivores (Erb and Reymond 2019; Jones et al. 2022). A major line of evasion of intoxication by animals is through chemosensory-mediated avoidance (Wooding et al. 2021; Xiao et al. 2022). However, behavioral responses are often inadequate to escape exposure, and toxic chemicals end up inside animals. Understanding how chemicals enter bodies (eg passive absorption and ingestion), the mechanisms underlying their movement between cells, tissues, and organs (eg diffusion and carrier/transporter proteins), whether they have unique targets or exert general effects (eg on cellular membranes), and how animals defend against such toxins (eg enhanced body shielding and toxin metabolism/excretion) are important issues spanning the fields of chemical ecology, neuroscience, physiology, and evolution.

The susceptibility and resistance mechanisms of insects to toxic chemicals is of particular interest, both because these processes reflect the insect perspective of their arms race with plant food sources (Erb and Reymond 2019; Jones et al. 2022) or venomous predators (Smith et al. 2013) and because humans have long used chemicals to combat insects that damage our agriculture or act as vectors of pathogens (Umetsu and Shirai 2020). The best-characterized natural and artificial insecticides are those that have a single, main cellular target (Sparks et al. 2020). For example, cardiac glycosides, produced by plants as defensive compounds by plants, exert their effect through inhibition of the sodium-potassium ATPase pump (Agrawal et al. 2012). Several insect orders have convergently evolved resistance to these toxins through amino acid substitutions in the α-subunit of this pump (Agrawal et al. 2012; Karageorgi et al. 2019). Plant-produced terpenoids are neurotoxic for insects through their antagonism of GABA receptors, and insects have evolved resistance through various mutations in this target receptor. Remarkably, similar mutations have arisen to confer resistance to the artificial insecticide dieldrin, which also targets these receptors (Guo et al. 2023). Indeed, many of the most widely used synthetic insecticides are agonists or antagonists of neuronal receptors or ion channels (Sparks et al. 2020). While these can be highly effective in killing insects, mutations conferring resistance on the target protein can be rapidly selected in insect populations (Carson 1962; Sparks et al. 2020). This phenomenon is most famously exemplified by dichlorodiphenyltrichloroethane (DDT), which prevents closure of voltage-gated sodium channels, an effect that has been circumvented in multiple insect orders through “knockdown resistance” (kdr) mutations in the corresponding genes (Busvine 1951; Dong 2007).

The mode of action and resistance mechanisms to many toxins remain less clear, typically where chemicals have more general effects on insect tissues, and resistance cannot be acquired through changes in a single gene. Such cases are commonly seen where species have adapted to toxic ecological niches and provide interesting models to explore broader toxicology mechanisms and evolution of resistance (Despres et al. 2007). For example, plant glucosinolates are potent defense compounds that chemically conjugate to exposed nucleophilic residues in diverse proteins leading to cellular and oxidative stress (Hopkins et al. 2009). The leaf-mining drosophilid *Scaptomyza flava* has evolved tolerance of such compounds, at least in part through duplication and neofunctionalization of glutathione-S-transferase-family detoxification enzymes (Gloss et al. 2014).

**Fig. 1.**
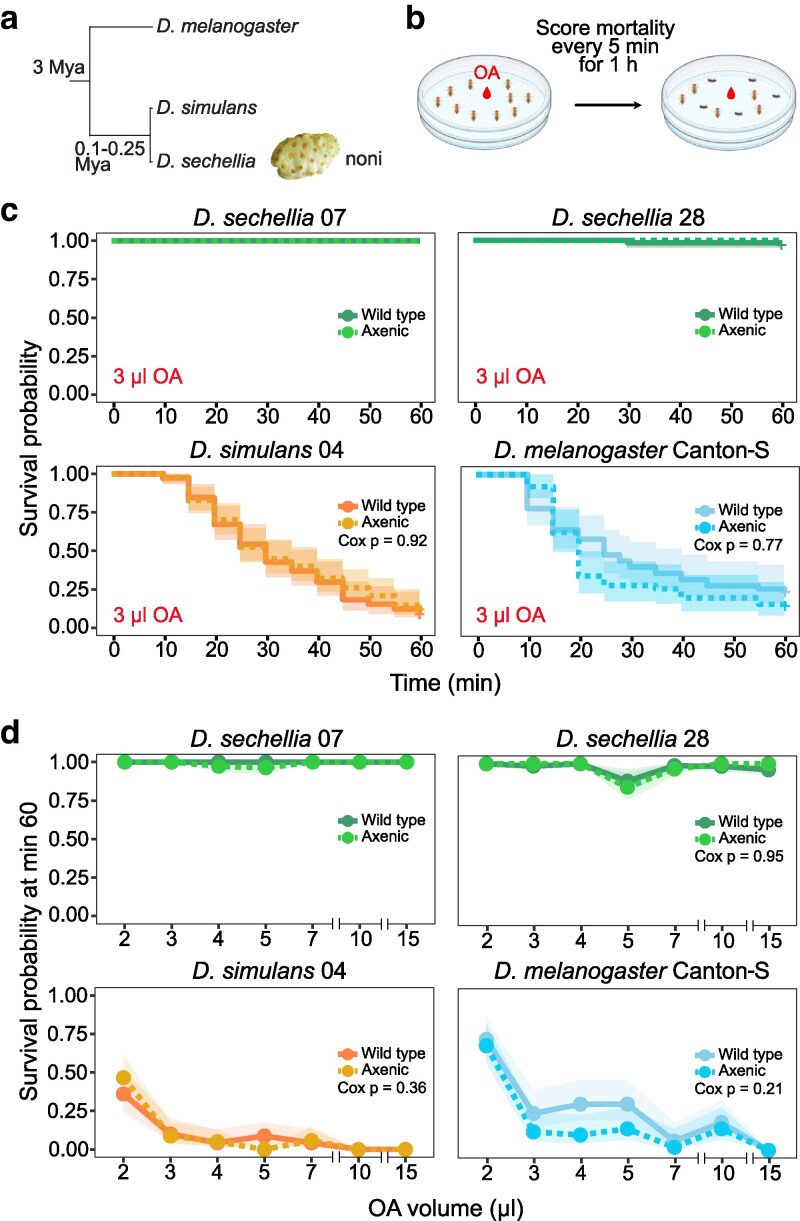
Octanoic acid resistance in axenic flies. a) Phylogeny of the *Drosophila* species used in this study. Mya, million years ago. b) Schematic of the octanoic acid (OA) resistance plate assay. c) Survival probabilities of axenic adult flies compared to wild-type strains to 3 μl OA over 60 min of exposure. *D. sechellia* 07 and 28 are 2 independent laboratory strains (see Supplementary Table 1). d) Survival probabilities of axenic adult flies compared to wild-type strains after 60 min of exposure to different volumes of OA. Each replicate for c and d consisted of 10 2- to 7-d-old female flies. Replicates, *n* ≥ 5; flies, *n* ≥ 50. The shaded areas represent the 95% confidence intervals. *P*-values are not reported when no mortality occurred for 1 or both groups (ie wild-type and axenic flies). Raw data are available in Supplementary File 1.

One long-recognized example of natural toxin resistance accompanying ecological specialization is the fruit fly *Drosophila sechellia*, which feeds and breeds exclusively on the acid-rich, toxic “noni” fruit of the shrub *Morinda citrifolia* (Farine et al. 1995; Jones 2005; Pino et al. 2009; Auer et al. 2021). The main noni toxin is thought to be octanoic acid (OA), which is lethal—through an unknown mechanism—to both very closely related drosophilid species, such as *Drosophila simulans* and *Drosophila melanogaster* (Fig. 1a) (Farine et al. 1995; Legal and Plawecki 1995), and more divergent insects, such as cockroaches, bees, and ants (Legal and Plawecki 1995). *D. sechellia*'s resistance to OA allows it to exploit ripe noni as a unique ecological niche (R'Kha et al. 1991; Legal et al. 1994; Amlou et al. 1997), thereby potentially escaping competition and parasitoidization (Salazar-Jaramillo and Wertheim 2021).

Pioneering attempts to analyze resistance to OA (or noni fruit) used quantitative trait locus (QTL) mapping in hybrids of *D. sechellia* and *D. simulans* at different life stages (Jones 1998, 2001; Huang and Erezyilmaz 2015). These efforts showed that at least 5 genomic loci contribute to resistance, including a major-effect region on chromosome arm 3R and additional loci on all other main chromosome arms (Jones 1998, 2001; Huang and Erezyilmaz 2015; Auer et al. 2021). While these regions are too large to pinpoint specific genes, subsequent fine mapping of the 3R QTL identified a ∼170 kb region, containing 18 genes. Testing of these candidates by RNAi in *D. melanogaster* identified 3 (*Osi6*, *Osi7*, *Osi8*) as modulators of OA resistance (Hungate et al. 2013; Andrade López et al. 2017; Lanno et al. 2019b), although if and how they contribute to OA resistance in *D. sechellia* await further investigation. Complementary studies employed comparative bulk transcriptomics of *D. sechellia* and other drosophilids in control and noni- or OA-exposed conditions to identify candidate toxin metabolism genes by virtue of their higher basal expression in *D. sechellia* and/or their induction by OA/noni presentation (Dworkin and Jones 2009; Lanno et al. 2017). Of a large number of candidates, the esterase Est-6 was implicated in OA tolerance through RNAi in *D. melanogaster* (Lanno et al. 2019a). Recently, we used experimental evolution of *D. simulans* and genome-wide CRISPR-based screening approaches in a *D. melanogaster* cell line to identify other genes contributing to OA resistance (Marconcini et al. 2025). Loss-of-function analysis of 2 of these in *D. sechellia*—the putative detoxification enzyme Kraken and the mitochondrial metabolic regulator Alkbh7—indicates that they contribute to OA resistance in this species (Marconcini et al. 2025), but it is clear that they are only part of the resistance mechanism. In this study we sought to identify additional candidate loci that contribute to OA susceptibility and resistance in drosophilids through complementary, cross-species genetic approaches.

## Materials and methods

### Drosophilid strains and culture

All drosophilid strains used in this study were reared on wheat flour/yeast/fruit juice medium at 25 °C under a 12 h light–dark cycle. Wild-type and transgenic strains are listed in Supplementary Table 1.

Axenic stocks were generated following standard protocols (Charroux and Royet 2020). In brief, we maintained stocks for 3 to 5 generations on standard medium supplemented with an antibiotic cocktail (50 µg/ml kanamycin (Applichem, CAS 5965-95-7), 50 µg/ml ampicillin (Applichem, CAS 69-52-3), 10 µg/ml tetracycline (Sigma Aldrich, CAS 64-75-5), and 5 µg/ml erythromycin (Carl Roth, CAS 114-07-8)) until germ-free. To assess the absence of fly-associated microbes, 10 flies were homogenized in 600 µl sterile PBS. From each homogenate, 100 µl were plated in triplicate on different microbiological media: Plate Count Agar (30 °C), MRS agar (37 °C), D-mannitol agar (30 °C), and LB agar (37 °C). Plates were incubated for 48 h to detect potential bacterial growth.

### OA resistance assay

OA resistance was assessed in plates or tubes essentially as described (Marconcini et al. 2025). In brief, for the plate assay, different volumes of OA were applied to the inside of the lid of a Petri dish (60 mm diameter, 15 mm height; Greiner Bio-One). Ten female flies were transferred to each plate after brief CO_2_ anesthesia for sorting. Flies were allowed to recover (typically 5 min), and mortality was recorded every 5 min for 60 min. Statistical analysis of the survival curves was conducted by fitting a mixed-effects Cox regression model as implemented in the R package coxme (Therneau 2024). Genotype was included as a fixed effect, and replicate dishes were modeled as a random effect. For axenic flies, the volume of OA was also included as a fixed effect.

For the tube assay, absorbent paper (Kimtech, 7552) was placed at the bottom of empty vials (25 mm diameter, 95 mm height, Milian SA), to which 1 ml of 3% D-glucose (neoFroxx, CAS 14431-43-7) solution was added, to prevent starvation and desiccation. Three microliters of OA (Sigma, CAS 124-07-2; >99% purity) were applied to the paper, and the vials were immediately sealed with cotton caps to minimize vapor loss. Flies (aged 1 to 7 d) were anesthetized with CO_2_, sexed, and transferred in groups of 25 males or 25 females per vial. Vials were placed upside down for 5 min to allow recovery and then maintained the right way round for 24 h at 25 °C under a 12 h light–dark cycle. Alive flies were counted at the end of the assay, and survival probability was calculated as the number of survivors/total number of flies. Control assays followed the same procedure, without the addition of OA. To test for sex differences in OA survival, we fitted a linear mixed-effects model with sex as a fixed effect and line as a random effect. Covariation between sexes was also examined by assessing the correlation between male and female median survival across lines.

For a subset of *D. simulans* strains, the tube assay was also used to assess resistance to 2 commercially available insecticides: the pyrethrin-based “Spruzit” AF (Neudorff, W-6670) (700 µl) and the fatty acid-based “Natural” (Andermatt Biogarten, W-6107) (100 µl). The volumes of these insecticides were chosen following pilot trials with a few *D. simulans* strains, representing doses that lead to a range of survival probabilities.

### Noni fruit resistance assay

Noni fruit was harvested from *M. citrifolia* plants cultivated in the University of Lausanne greenhouses. Noni pulp purée was prepared by homogenizing 5 ripe fruits and stored at −20 °C until use. Resistance was assessed in the tube assay as described above, replacing OA and the tissue paper soaked with glucose with 2 g of thawed noni pulp purée. A previous study indicated that noni contains 3.06 g OA/kg fruit (Pino et al. 2009); assuming an equivalent quantity in our fruits, 2 g noni pulp should contain 6.73 µl OA, approximately 2-fold higher than the amount of pure OA used. For the tested *D. melanogaster* and *D. simulans* lines, the correlation between median OA resistance and median noni resistance across lines was assessed using a linear model fitted with the lm() function in R 4.0.3.

### Genome-wide association studies

*D. melanogaster*: For the *D. melanogaster* Genetic Reference Panel (DGRP), we downloaded the binary files from the DGRP2 website (https://www.re3data.org/repository/r3d100011826). The median survivability scores to OA were used to test for genotype–phenotype association. We fitted a linear model in Plink2.0 using the following parameters: --glm hide-covar --quantile-normalize --variance-standardize --geno 0.2 --maf 0.05. We controlled for population structure by adding the --covar parameter using the first 20 PCs obtained with --pca 20. We also controlled for known inversions (ie *In(2L)t*, *In(2R)NS*, *In(2R)Y1*, *In(2R)Y2*, *In(2R)Y3*, *In(2R)Y4*, *In(2R)Y5*, *In(2R)Y6*, *In(2R)Y7*, *In(3L)P*, *In(3L)M*, *In(3L)Y*, *In(3R)P*, *In(3R)K*, *In(3R)Mo*, *In(3R)C*) and *Wolbachia* infection modeling them as covariates. The analysis was conducted both on the combined dataset of males and females, as well as separately for each sex. In total 1,891,456 variants were tested.

*D. simulans*: The raw genomic data were downloaded from SRA (SRP075682), aligned to the *D. simulans* reference genome (NCBI GCF_016746395.2) using bwa-mem (Li 2013), and the results were sorted and compressed using samtools (Danecek et al. 2021). Duplicate reads were then removed with Picard MarkDuplicates (https://broadinstitute.github.io/picard/). Variants were identified using GATK HaplotypeCaller (McKenna et al. 2010). We filtered variants with quality lower than 30 with vcftools 0.1.17 (Danecek et al. 2021). Binary input files for the genome-wide association studies (GWAS) analysis were generated with Plink2.0 (Purcell et al. 2007; Purcell 2015). We excluded 2 lines from the analysis: line 235, for which no sequencing data were available, and line 237, due to evidence of potential contamination in the NGS data. The median survivability scores to OA were used to test for genotype–phenotype association. We fitted a linear model in Plink2.0 following the same procedure as for the DGRP, resulting in testing of 3,262,537 variants. Linkage disequilibrium maps of focal regions were generated with LDBlockShow using the solid spine method based on *D*′ statistics (Dong et al. 2021).

Plink input files for GWAS analyses are provided in Supplementary File 5.

### Transcriptome-to-phenotype association

Whole-fly, bulk RNA-seq data for DGRP lines (Huang et al. 2015) were downloaded from the DGRP2 website (https://www.re3data.org/repository/r3d100011826), and mean expression across replicates was computed for each transcript. We fitted a linear model between the median survivability scores to OA and each transcript, as in Battlay et al. (2018) and Green et al. (2019), for the 161 lines for which both phenotypic and transcriptomic data are available. A *P*-value significance threshold of 1 × 10^−3^ was applied, following Battlay et al. (2018). Analyses were conducted both on the combined dataset of males and females, as well as separately for each sex. The results were visualized using a Manhattan plot, with each analyzed transcript mapped to the genomic position of its corresponding gene.

### Protein structure prediction

Three-dimensional structure predictions of Bark variants (ie with I2114 or V2114, within the longest-encoded protein isoform) were generated using the implementation of AlphaFold2 (Jumper et al. 2021) on the ColabFold platform (https://colab.research.google.com/) (Mirdita et al. 2022), using default parameters. Five ranked models were generated for each sequence, and the top-ranked structure, based on the predicted local distance difference test (pLDDT) score, was selected for further analysis. The variant protein structures were aligned and visualized in ChimeraX.

## Results

### OA susceptibility and resistance in drosophilids are independent of the microbiome

The high resistance of *D. sechellia* to OA/noni has been assumed to reflect genetic adaptation of this species (Jones 1998; Huang and Erezyilmaz 2015). However, resistance of various insect species to several toxic chemicals has been linked to the microbiome (Brown et al. 2021 ; Malook et al. 2025), through presumed microbial functions in chemical detoxification or modulation of host gene expression and physiology. Comparative studies of drosophilid microbiota indicate that *D. sechellia* collected from noni fruit has a distinctive microbiome, dominated by a single *Lactobacillales* taxon, but otherwise an extremely low bacterial community richness and evenness (Chandler et al. 2011; Heys et al. 2021). Moreover, when *D. melanogaster* was fed food containing the *D. sechellia* microbiome, it lost its normal aversion to OA (Heys et al. 2021), raising the possibility that the *D. sechellia* microbiota can influence host behavior.

We therefore first investigated whether the microbiome of *D. sechellia* contributes to this species' resistance to OA by generating axenic flies, through culture on a cocktail of antibiotics (see *Materials and methods*). Given the low diversity of the *D. sechellia* microbiome and the observations that microbes can sometimes increase toxicity through metabolic “activation” (Peterson 2024), we also generated axenic *D. simulans* and *D. melanogaster*. These flies, as well as untreated controls, were tested for OA resistance in a “plate assay” (Jones 1998; Marconcini et al. 2025), in which mortality of flies exposed to a droplet of OA is monitored over the course of 1 h (Fig. 1b). As expected, when exposed to 3 μl OA (comparable to a dose in noni, see *Materials and methods*), the 2 *D. sechellia* strains showed almost no mortality, whereas both *D. simulans* and *D. melanogaster* had very few survivors by the end of the experiment (Fig. 1c). Importantly, no differences in survival probability were observed between axenic and control strains for any species (Fig. 1c). We then extended the assay to a broader range of OA volumes and found no evidence of differences in survivorship between axenic and control flies (Fig. 1d). These results suggest that the microbiomes of these drosophilid species do not make a measurable contribution to OA resistance, reinforcing the idea that this trait is primarily genetically determined and distinct from microbiome-mediated effects on OA preference or aversion observed at nonlethal doses (Heys et al. 2021).

### Phenotypic variation in OA resistance across genetically diverse *D. melanogaster* and *D. simulans*

One effective approach to identify the genetic basis of complex traits is to examine the relationship between genetic and phenotypic variation, as implemented in genome-wide association studies (GWAS). However, GWAS analyses require both substantial standing genetic variation and adequate statistical power, which is largely determined by the number of available genotypes or strains. These requirements are difficult to meet in *D. sechellia*, as no large strain panel exists and the species is over an order of magnitude less polymorphic than other members of the *D. melanogaster* subgroup (Legrand et al. 2009). Commensurately, OA resistance appears similar across tested laboratory and wild-caught strains (Shahandeh et al. 2026). A study of a small number of wild-type *D. melanogaster* and *D. simulans* strains has revealed the existence of intraspecific variation in sensitivity to OA (Supplementary Fig. 1a; Colson 2004; Andrade López et al. 2017). Given the phylogenetic proximity of these species to *D. sechellia* (Fig. 1a), we reasoned that we might uncover novel loci contributing to OA resistance by taking advantage of the substantial genetic diversity within large collections of sequenced, isogenic strains of *D. melanogaster* (the DGRP (Mackay et al. 2012), originating from a fruit market in Raleigh, North Carolina) and *D. simulans* (Signor et al. 2018) (originating from the Zuma organic orchard, California).

**Fig. 2.**
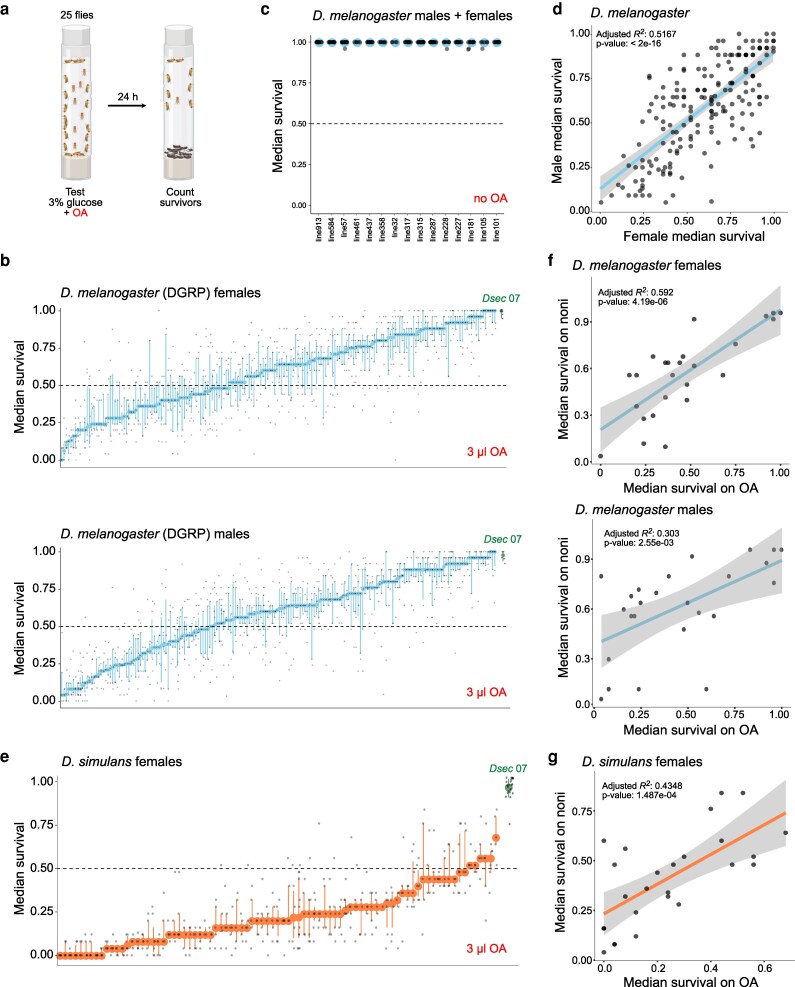
Phenotypic characterization of OA resistance in *D. melanogaster* and *D. simulans*strains. a) Schematic of the OA resistance tube assay. b) Representation of OA resistance of the *D. melanogaster* (DGRP) strains for females and males. Strains are arranged on the *x* axis by increasing median survival (*y* axis). Blue dots are the median values, blue whiskers are the inter-quantile ranges, and gray dots are the individual replicates. Genotype ordering on the *x* axis is independent for the 2 sexes. Raw data are available in Supplementary File 1. c) Median survival of a subset of *D. melanogaster* strains in the absence of OA. d) Male/female survival correlations based on the median values from b. The shaded area represents the 95% confidence interval. e) Representation of OA resistance of the *D. simulans* strains for females. Strains are arranged on the *x* axis by increasing median survival (*y* axis). Orange dots are the median values, orange whiskers are the inter-quantile ranges, and gray dots are the individual replicates. Raw data are available in Supplementary File 1. f and g) OA/noni fruit survival correlation for a subset of 25 DGRP strains (f) and 25 *D. simulans* strains (g). The shaded area represents the 95% confidence interval. Raw data are available in Supplementary File 2.

To analyze OA resistance in these large panels, we used a simpler “tube assay” (Amlou et al. 1997; Colson 2004; Marconcini et al. 2025), in which ∼25 flies were placed in a culture vial containing a tissue paper spotted with 3 µl of OA in 3% glucose solution, and scored viability after 24 h (Fig. 2a). In previous studies of various non-*D. sechellia* drosophilids (Amlou et al. 1997; Colson 2004; Marconcini et al. 2025), such a quantity of OA resulted in lethality of approximately half of the animals, making it an effective dose for screening purposes.

Using this assay, we analyzed resistance to OA in males and females of 186 *D. melanogaster* lines from the DGRP (Supplementary Table 1). Survival to OA varied remarkably widely across the strains in both sexes, with median survival ranging from nearly 0 to 1, reaching *D. sechellia*-like levels of resistance (at least under these conditions) (Fig. 2b). The high intrastrain variability is concordant with previous toxicological studies with OA (Amlou et al. 1997; Colson 2004). In control assays without OA, using a subset of lines spanning the range of survival probabilities, we found survival of essentially all flies regardless of strain or sex (Fig. 2c), indicating that the mortality observed under experimental conditions is attributable to OA exposure. Survival probabilities were strongly correlated between sexes (Fig. 2d), and no significant sex differences were revealed in a linear mixed model including sex as a fixed effect and strain as a random effect (*β* = −0.0128 ± 0.0138, *t* = −0.924, df = 185, *P* = 0.357). To assess a potential effect of age, which might affect the degree of resistance through the maturation state of cuticular hydrocarbons (Kuo et al. 2012), we examined OA resistance of a subset of strains aged between 1 and 2 d or 3 and 7 d post eclosion. No differences were observed in any of the tested strains (Supplementary Fig. 1b).

We performed a similar screen of a panel of *D. simulans* strains (Signor et al. 2018) (Supplementary Table 1). As in *D. melanogaster*, no sex differences were observed in a small subset of lines (Supplementary Fig. 1c), leading us to restrict full screening to females. Across the 84 tested lines, we observed a wide range of survivability, from 0 to 0.68 median survival probability (Fig. 2e). In control assays in the absence of OA, essentially complete survival of all tested lines was observed (Supplementary Fig. 1d).

Together these screens reveal striking phenotypic diversity in OA resistance in both *D. melanogaster* and *D. simulans* lines. The broad distribution of survival probabilities across the panels indicates that susceptibility/resistance can be shaped by numerous genetic factors.

### OA resistance correlates with resistance to noni but not other toxins

To evaluate the potential ecological relevance of OA resistance observed in these *D. melanogaster* and *D. simulans* panels, we examined 25 *D. melanogaster* and 25 *D. simulans* strains—spanning the observed ranges of OA survivability—for resistance to noni pulp. Survival on noni strongly correlated with survival on OA in both sexes of *D. melanogaster* (Fig. 2f) and in *D. simulans* (Fig. 2g). These results confirm the central contribution of this chemical to fruit toxicity and, by extension, imply that the genetic determinants of susceptibility/resistance to OA are potentially relevant to underlie susceptibility/resistance to noni.

The DGRP has been extensively characterized for a multitude of phenotypes, including resistance to diverse insecticides, such as organophosphates, pyrethroids, and neonicotinoids (Battlay et al. 2016, 2018; Denecke et al. 2017; Najarro et al. 2017; Schmidt et al. 2017; Duneau et al. 2018; Green et al. 2019). We assessed correlations of resistance to OA and these other toxins using DGRPool (Gardeux et al. 2023), but no significant associations were detected. Similarly, in more limited testing of *D. simulans* survival on pyrethrin-based and fatty acid-based insecticides, we did not detect any significant correlation between the degree of resistance of different lines to these chemicals and OA (Supplementary Fig. 1e). These findings imply that the mechanistic basis of OA susceptibility and resistance is distinct from that of other insecticides.

Broader surveying of OA resistance with >500 other traits in DGRPool revealed a weak but significant correlation with alcohol sensitivity (suggestive of partially overlapping toxicity mechanisms) (Supplementary Table 2); other correlated traits, including male aggression and average lifespan, are harder to explain.

Finally, we tested for correlation between OA resistance and *Wolbachia* infection. *Wolbachia*-infected *D. melanogaster* showed slightly higher median survival (estimate = 0.083, *P* = 0.020), but the effect was very small and explained less than ∼3% of the variation in OA resistance (adj. *R*² = 0.024). These results are consistent with our axenic fly assays (which should also lack *Wolbachia* infection (Shropshire 2024)), further supporting the conclusion that the microbiome plays little to no role in this phenotype.

### Genome-wide association studies of OA resistance

**Fig. 3.**
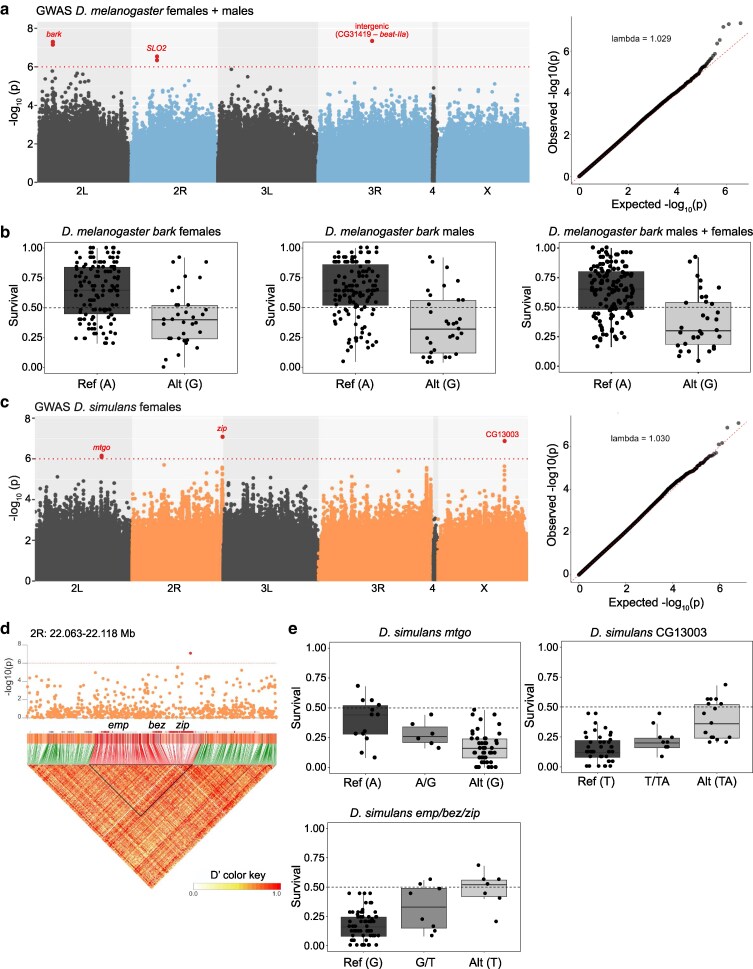
Genome-wide association analysis of OA resistance in *D. melanogaster* and *D. simulans* strains. a) Left: Manhattan plot of the GWAS of OA resistance, based on median survival of males and females combined. Each dot represents a SNP, plotted on the *x* axis according to its genomic position. The *y* axis shows −log_10_(*P*) values for the association between genotype and OA resistance. The red dotted line marks a conservative arbitrary threshold at *P* ≤ 1 × 10^−6^. SNPs exceeding this threshold are highlighted in red. Right: quantile–quantile (Q–Q) plot showing the observed distribution of association test statistics versus the expected distribution under the null hypothesis of no association. Raw data are available in Supplementary File 3. b) Boxplots of median OA survival of the tested strains containing the reference (Ref) or alternative (Alt) alleles for the most significant SNP in *bark* for females, males, and combined sexes. c) Same as in a but for *D. simulans* females. d) Zoomed-in Manhattan plot of the 2R region (2R: 22.062 to 22.118 Mb) around the significant SNP with underlying linkage disequilibrium (LD) structure. LD is represented as a heatmap (*D*′ values, ranging from white to red), with genes encompassed within the significant LD block (black triangle) highlighted in red. e) Boxplots showing median OA survival across tested strains carrying the reference (Ref), heterozygous, or alternative (Alt) alleles for the most significant SNPs identified in c. The title of each plot indicates the gene(s) encompassed by the corresponding significant SNP.

To investigate the genetic basis of the observed variation in OA resistance, we tested for associations between median resistance to OA (Supplementary File 1) and genetic variants segregating in the respective species' panels. For the DGRP, GWAS was performed separately for males and females (Supplementary Fig. 2a) as well as in a combined analysis (Fig. 3a). Only 1 SNP was identified across all 3 analyses (Fig. 3a and Supplementary Fig. 2a); this SNP falls in the coding sequence of the gene *bark beetle* (*bark*, also known as *anakonda*) on chromosome 2L, resulting in an amino acid substitution (I2114V), as discussed further below. The presence of the alternative allele significantly reduced OA resistance in both male and female flies (Fig. 3b). Beyond the sole SNP common to the GWAS of males, females, and combined sexes (in *bark*), several other significant SNPs were found only in the male and combined analyses and were not considered further in this work.

GWAS of the *D. simulans* panel identified 3 peaks (Fig. 3c). A small peak on 2L falls on the gene *miles to go* (*mtgo*), while the peak on the X chromosome lies in CG13003. The main peak on chromosome 2R is located in an exon of *zipper* (*zip*). However, this locus is part of a 22.5 kb linkage disequilibrium block comprising 2 additional genes, *epithelial membrane protein* (*emp*), and *between emp and zip* (*bez*) (Fig. 3d). Because these genes are in strong linkage disequilibrium, it is not possible to determine whether the association with the phenotype is driven by 1 specific gene or by multiple genes within this block. Unlike the DGRP, this panel is not fully isogenic or fixed at the loci examined, allowing heterozygous genotypes to be observed. For each of these loci, alternative alleles showed additive effects on OA survivability (Fig. 3e), with heterozygous lines exhibiting intermediate phenotypes between the 2 homozygous genotypes. The identified variants all fall in noncoding regions except for the one in *zip*, which causes a synonymous mutation in position R445. The effect of these variants is unclear, and we speculate that their role is regulatory or that the real causative SNPs were not picked up by our analysis.

### Expression, sequence, and functional analysis of candidate OA resistance genes

**Fig. 4.**
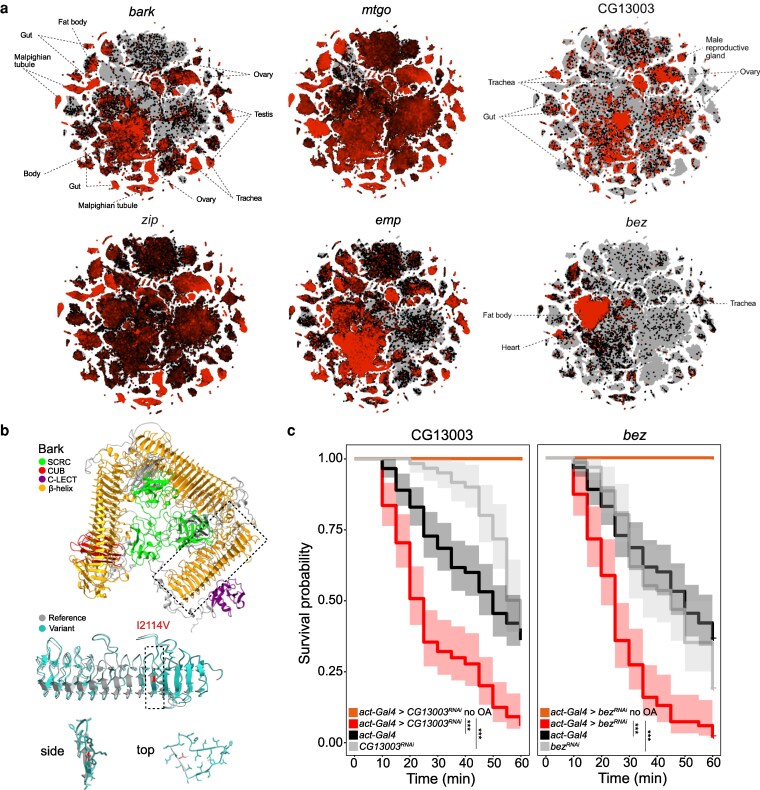
Expression, protein modeling, and functional validation of OA resistance genes in *D. melanogaster.* a) Expression profiles of candidate genes from the Fly Cell Atlas (10× stringent dataset). Relative expression levels are depicted on the default black-to-red scale from the SCope interface, with labels marking tissues in which expression is enriched. b) Top: predicted structure of Bark (based on the *D. melanogaster* sequence) carrying the reference variant (I2114). The structure shows the tripartite organization of bark: The β-helix domains form the characteristic β-barrel architecture. Middle: detailed view of the β-helix domain, showing the predicted arrangement of β-helix repeats. The model compares the reference (I2114, gray) and variant (V2114, blue) structures. The location of residue 2114 is marked in red. Bottom: magnified views (side and top) of the β-helix repeat carrying I2114V (red). c) OA survival curves for and CG13003*^RNAi^* and *bez^RNAi^* flies and the corresponding controls. Each replicate consisted of 10 2- to 7-d-old female flies. Replicates, *n* ≥ 6; flies, *n* ≥ 60. The shaded area represents the 95% confidence interval. Genotypes: *act-Gal4/+;UAS*-*CG13003^RNAi^/+*, *act-Gal4/+*, *UAS-CG13003^RNAi^/+*, *act-Gal4/+;UAS*-*bez^RNAi^/+*, *act-Gal4/+*, *UAS-bez^RNAi^/+*. Raw data are available in Supplementary File 1.

We proceeded to examine the candidate genes using available information from expression (Fly Cell Atlas (Li et al. 2022), Fly Atlas 2 (Krause et al. 2022)) and functional studies in *D. melanogaster* (from FlyBase (Öztürk-Çolak et al. 2024)) (Fig. 4, a and b, and Supplementary Fig. 2b).

The sole consistent candidate from the *D. melanogaster* GWAS, *bark*, encodes a large (>3,000 amino acids) type I transmembrane protein. Based on AlphaFold predictions, the Bark extracellular domain is composed of 3 repeats of a scavenger receptor cysteine-rich domain and β-helix repeats and also contains CUB and C-type lectin domains (Fig. 4b). *bark* is broadly expressed in *D. melanogaster*, including the digestive, renal, excretory, and reproductive systems (Fig. 4a), with the greatest tissue enrichment in the midgut (Supplementary Fig. 2b). Bark has an important function in the maturation of septate junctions between epithelial cells in various tissues (Byri et al. 2015; Hildebrandt et al. 2015). Such junctions have a critical role in cell–cell adhesion and in the prevention of diffusion of small molecules through the paracellular space. The variant amino acid position associated with OA resistance (I2114V) is located within one of the β-helix domains of the third repeat. To assess the potential effect of this variant on protein structure, we generated structural models of this domain using both the reference (I2114) and variant (V2114) sequences. Superposition of these models did not reveal a noticeable difference between them (Fig. 4b); however, we cannot exclude the possibility that this amino acid variant leads to a subtle conformational change and/or has an effect on protein–protein interactions of Bark.

Of the candidates emerging from the *D. simulans* GWAS, *mtgo* encodes a member of the fibronectin type III superfamily of cell adhesion proteins (Syed et al. 2019). In *D. melanogaster*, this gene displays near-ubiquitous expression; consistently loss-of-function mutations lead to pupal lethality due, in part, to a developmental requirement at neuromuscular junctions (Syed et al. 2019).

CG13003 is uncharacterized and lacks recognizable protein domains. However, it contains an N-terminal signal sequence, suggesting that it is secreted, and is enriched in proline and serine residues (each >10% primary sequence). Such properties are reminiscent of extracellular matrix proteins, such as mucins (Syed et al. 2008). In *D. melanogaster* larvae, CG13003 expression is most highly enriched in tracheae (Supplementary Fig. 2b), which is also observed in adults, together with expression in gut epithelia and reproductive tissues (Fig. 4a). We speculate that the encoded protein contributes to the formation of an extracellular barrier that protects tissues from OA toxicity.

Within the linkage disequilibrium block on chromosome 2R, all 3 genes (*zip*, *emp*, and *bez*) exhibit properties of potential relevance for OA resistance. *zip* is very broadly expressed and encodes a nonmuscle myosin heavy chain required for regulating the actin cortical network in many cellular and tissue contexts. Within this general role, however, *zip* interacts with septate junction-associated proteins and contributes to actomyosin contractility that can influence junctional organization (Holland et al. 2025), hinting at the possibility that Zip and Bark affect OA resistance through a related mechanism. *emp* and *bez* both encode members of the CD36 family of transmembrane scavenger receptors, which have diverse ligands and biological roles (Silverstein and Febbraio 2009; Pepino et al. 2014). *emp* is broadly expressed in epithelial tissues, including tracheae, and is an essential gene that is best characterized for its role in morphogenesis of the airways (Pinheiro et al. 2023); as for CG13003, this gene might be pertinent for OA resistance should the tracheal system be an entry point for OA into the body. Of particular interest, however, is Bez, which is most prominently expressed in the fat body, where it functions as a fatty acid transporter involved in lipid export from this organ (Carrera et al. 2024). The insect fat body is a key tissue for chemical storage and detoxification (Arrese and Soulages 2010), raising the possibility that altered function of Bez contributes to OA resistance through enhanced sequestration in this organ.

Manipulation of many of these candidate genes to test their contribution to OA resistance is complicated by their essentiality for animal viability (Öztürk-Çolak et al. 2024). We therefore focused on CG13003 and *bez*, both because these are the most selectively expressed of the candidates (Fig. 4a) and because these genes were also linked to increased OA resistance in an experimental evolution experiment in *D. simulans* (Marconcini et al. 2025). Transgenic RNAi knockdown of these genes did not affect survival or lead to obvious morphological or behavioral defects. However, when tested for resistance to OA, both CG13003*^RNAi^* and *bez^RNAi^* animals displayed a significant reduction in survivability compared to control genotypes (Fig. 4c). These results suggest that the encoded proteins contribute to conferring resistance, rather than susceptibility, to this toxin. Although these data support the promise of the GWAS in identifying functionally relevant genes, future studies will be required to assess whether these and other genes are also relevant for OA resistance in *D. sechellia* and to elucidate their precise mechanism of action.

### Transcriptome-to-phenotype association identifies additional candidate OA resistance genes

**Fig. 5.**
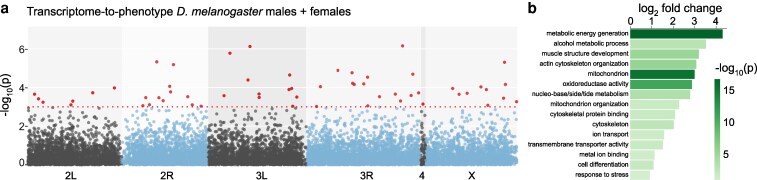
Transcriptome-to-phenotype associations for OA resistance in DGRP strains. a) Manhattan plot of the transcriptome-to-phenotype association analysis in the DGRP, based on median expression values combining males and females. Each gene is represented as a single dot positioned along the *x* axis according to its genomic location. The *y* axis shows the −log_10_(*P*) for the association between expression and OA resistance. The red dotted line marks an arbitrary significance threshold of *P* ≤ 1 × 10^−3^. Transcripts exceeding this threshold are highlighted in red.b) PANGEA gene ontology analysis of the significant genes identified in a using the curated *Drosophila* GO subsets. Raw data are available in Supplementary File 4.

While GWAS is powerful for pinpointing SNPs associated with a trait, a transcriptome-to-phenotype study allows the identification of differences in gene expression that might be functionally relevant. The availability of whole-fly, bulk RNA-sequencing datasets for the DGRP (Huang et al. 2015) has enabled transcriptome-to-phenotype analyses on this panel for various insecticide resistance traits, identifying several detoxification-related genes (Battlay et al. 2016; Denecke et al. 2017; Green et al. 2019). Profiting from this dataset, we fitted a linear model regressing median resistance to OA to the mean expression of 18,140 transcripts. This analysis yielded 52, 16, and 75 candidate transcripts for males, females, and the combined dataset, respectively, that display an association between expression level and OA resistance (Fig. 5a). We could not find common hits with our GWAS analyses. Gene ontology analysis using PANGEA (Hu et al. 2023) revealed enrichment for a range of terms, including metabolic processes (eg the NADH dehydrogenase ND-75 and the cytochrome P450 Cyt-c1), mitochondrial function (eg ATP synthase subunit D and Superoxide dismutase 2), and cytoskeleton organization (eg Act88F and Tm2) (Fig. 5b). The enrichment of these pathways matches well with those identified in complementary genetic analyses of OA resistance through experimental evolution and CRISPR-based loss-of-function screens in cell lines (Marconcini et al. 2025).

## Discussion

The evolution of insect resistance to toxins is often linked most strongly with mutations in genes encoding target proteins, with striking examples of parallel or convergent evolution between different insect species exposed to natural or artificial toxins that have the same target (Busvine 1951; Dong 2007; Guo et al. 2023). Resistance to the toxin OA appears to be fundamentally different. This trait was historically first-recognized in insects because of the striking, high resistance exhibited by *D. sechellia*, which is a critical adaptation to its specialized ecological niche (R'Kha et al. 1991; Legal et al. 1994; Amlou et al. 1997; Auer et al. 2021). Mapping studies revealed a complex genetic architecture of *D. sechellia*'s OA resistance (see *Introduction*). More recently, a strain of *Drosophila yakuba*—a distant relative within the *D. melanogaster* subgroup of drosophilids—was found to exhibit similar ecological specialization on noni fruit and displays higher OA resistance than the parental species (Yassin et al. 2016). Using a genome-wide scan of targets of natural selection across *D. yakuba* populations, several loci associated with this recent specialization on noni were identified, but these displayed only limited overlap with genomic regions identified in *D. sechellia* species (Yassin et al. 2016). In this work, we show that even different inbred strains of *D. melanogaster* and *D. simulans* exhibit large variation in OA susceptibility/resistance and capitalize on such phenotypic variation to identify, through association studies, several novel candidate genes contributing to this trait.

Integrating the genes identified in this work with those that emerged from previous studies of OA resistance in various drosophilid species (Table 1 and *Introduction*) reinforces the evidence for the contribution of several pathways to this phenotype. The nature of the encoded proteins and expression patterns points to a complex interplay of multiple tissues and molecular processes underlying resistance to OA. Such breadth suggests that OA does not have a singular cellular or molecular target, but might affect a basic cellular process, an idea supported by the toxicity of OA to cultured insect cell lines (Kaczmarek et al. 2022, 2024; Marconcini et al. 2025), yeast (Mota et al. 2024), and bacteria (Lennen et al. 2011). The candidate resistance genes seem less likely to be a target of OA but rather contribute to a variety of defense mechanisms, including cuticular and epithelial barrier functions, gut/renal system chemical detoxification, lipid transport, and general metabolic activity. Identification of Bark from our *D. melanogaster* GWAS is of note, as it highlights a novel defense mechanism of insects against toxic chemicals. Oral insecticides are thought to have to cross the midgut epithelium to be able to exert their toxic effects (Denecke et al. 2018), but there has been almost no previous consideration for how natural variation in the integrity of this internal tissue barrier—analogous to the external cuticular barrier—might determine susceptibility or resistance to toxins (Chen et al. 2023). Examination of the contributions of other genes involved in septate junction formation to resistance to OA, as well as other toxic chemicals, will be of interest.

**Table 1.** Summary of properties of candidate genes underlying OA resistance.

Gene	Protein domains	Putative/known cellular role(s)	Notable tissue expression	Species	Stage	Methods/supporting data	References
*Alkbh7* (CG14130)	α−Ketoglutarate-dependent dioxygenase	Mitochondrial RNA demethylation; programmed necrotic cell death; fatty acid metabolism	Ubiquitous	*Dmel*/*Dsim*/*Dsec*	Adult	Experimental evolution, CRISPR screen, RNAi, KO, expression analyses	Marconcini et al. (2025)
*bark*	SCRC, CUB, C-LECT, β-helix	Septate junction maturation	Broad	*Dmel*	Adult	GWAS	This study
*bez*	CD36	Fatty acid transport	Fat body	*Dsim*/*Dmel*	Adult	GWAS, RNAi	Marconcini et al. (2025), this study
CG13003	Mucin-like?	Unknown	Trachea	*Dsim*/*Dmel*	Adult	GWAS, RNAi	Marconcini et al. (2025), this study
*emp*	CD36	Fatty acid transport	Ubiquitous	*Dsim*	Adult	GWAS	This study
*Est-6*	Type-B carboxylesterase/lipase	Metabolic detoxification	Broad	*Dmel*	Adult	RNA-seq and RNAi	Lanno et al. (2019a)
*gce*	Myc-type, bHLH PAS	Juvenile hormone receptor	Ubiquitous	*Dsec* × *Dsim*	Larva	QTL	Huang and Erezyilmaz (2015)
				*Dyak*	Adult	Population branch excess	Yassin et al. (2016)
*GST-E* genes	Glutathione S-transferase	Metabolic detoxification	Broad	*Dsec*	Adult	Microarray	Dworkin and Jones (2009)
				*Dyak*	Adult	Population branch excess	Yassin et al. (2016)
*kraken*	Serine hydrolase	Metabolic detoxification	Excretory system	*Dmel*/*Dsim*/*Dsec*	Adult	Experimental evolution, CRISPR screen, RNAi, KO, expression analyses	Marconcini et al. (2025)
*mtgo*	Fibronectin type III	Neuromuscular junction branching and growth	Ubiquitous	*Dsim*	Adult	GWAS	This study
*Osi6*, *Osi7*, *Osi8*	DUF1676	Endosomal-localized; regulation of cuticle secretion/shaping	Epithelia, Trachea	*Dsec* × *Dsim*	Adult	Introgression with CAPS markers	Hungate et al. (2013)
				*Dyak*	Adult	Population Branch Excess	Yassin et al. (2016)
				*Dsec*	Larva, adult	RNA-seq	Lanno et al. (2017); Lanno et al. (2019b)
				*Dmel*	Larva, adult	RNAi	Andrade López et al. (2017); Lanno et al. (2019b)
Serine protease genes	Peptidase S1 family serine proteases	Detoxification	Reproductive organs, epithelia	*Dyak*	Adult	Population branch excess	Yassin et al. (2016)
*Tweedle* genes	DUF243	Chitin-binding, cuticle development	Epithelial, trachea	*Dsec* × *Dsim*	Larva	QTL	Huang and Erezyilmaz (2015)
				*Dyak*	Adult	Population branch excess	Yassin et al. (2016)
*zip*	Nonmuscle myosin 2 heavy chain	Cortico-actin dynamics	Ubiquitous	*Dsim*	Adult	GWAS	This study

The limited overlap in the specific genes identified between individual studies (Table 1) reinforces the idea of no singular major-effect gene contributing to OA resistance. One explanation for the lack of common hits between the present *D. melanogaster* and *D. simulans* GWAS, and with previous investigations, is that GWAS can only recover associations from the finite variation segregating in a given panel of strains. Thus, different starting populations will yield different sets of candidate genes. We also note that there is only partial overlap between the *D. simulans* GWAS with genetic variants identified in our previous experimental evolution of *D. simulans* populations (Marconcini et al. 2025), despite the base populations being initially generated from the same lines profiled here. The failure to identify, for example, *kraken* in the GWAS might be because of insufficient variability of this gene across the lines, a small effect size, or a detectable contribution of this gene only in a particular genetic background (of both strain and species). More generally, the contributions of different genes to OA susceptibility/resistance are also likely to depend upon the life stage, the dose of this chemical, and the method of exposure, one or more of which factors vary between different studies.

The limited overlap of specific genes between studies in different strains/species also suggests there are many possible paths toward OA resistance. It is nevertheless notable that standing genetic variation in populations of *D. melanogaster* and *D. simulans*—both isolated from restricted geographic localities—is sufficient to encode a broad phenotypic range in resistance to OA. This observation hints at the possibility that OA resistance in *D. sechellia* also emerged, at least initially, from genetic variants already present in the ancestor of *D. sechellia* and *D. simulans* (and perhaps also *D. melanogaster*) rather than through *de novo* mutations of large-effect size.

In summary, while previous analyses of OA resistance have emphasized detoxification and cuticular defense, our findings point to additional layers of resistance involving internal epithelial barriers and metabolism. Given the evident genetic complexity of this superficially simple trait, it is useful to combine the results of distinct approaches—each with their advantages and disadvantages—to identify candidate genes underlying this phenotype. Further work will be required to determine whether genetic associations reflect causal requirements, both in laboratory inbred strains and those in nature, and the precise site and mechanism of action of the encoded proteins.

## Supplementary Material

jkag032_Supplementary_Data

## Data Availability

The authors affirm that all data necessary for confirming the conclusions of the article are present within the article, figures, tables, and supplementary files. Scripts to reproduce the axenic flies' plots and statistics, as well as the GWAS and transcriptome-to-phenotype analyses, are hosted on the public GitHub repository (https://github.com/mmarconc/Drosophila_GWAS_ms). Raw data for OA resistance in axenic flies are available in Supplementary File 1. Raw data for the phenotypic characterization of OA resistance in *D. melanogaster* and *D. simulans* strains are available in Supplementary File 2. The results of the GWAS analyses are available in Supplementary File 3. The results of the transcriptome-to-phenotype analysis are available in Supplementary File 4. Input files to reproduce the GWAS analyses are available in Supplementary File 5. Wild-type and transgenic strains are listed in Supplementary Table 1. The top 10 phenotypic correlations with OA resistance in the DGRP are listed in Supplementary Table 2. Supplementary Files 3 and 5 are located at GSA FigShare: https://doi.org/10.25387/g3.31158451. Supplemental material available at G3 online.
