# Supplementary material for: Genome-wide association studies identify new candidate genes and tissues underlying resistance to a natural toxin in drosophilids
Source: G3 (Bethesda). 2026 Feb 16;16(4):jkag032. doi: 10.1093/g3journal/jkag032 (PMC13042304; doi:10.1093/g3journal/jkag032)
Supplement: jkag032_Supplementary_Data [file jkag032_supplementary_data.zip › Figure_S1_G3-2026-406569.pdf]

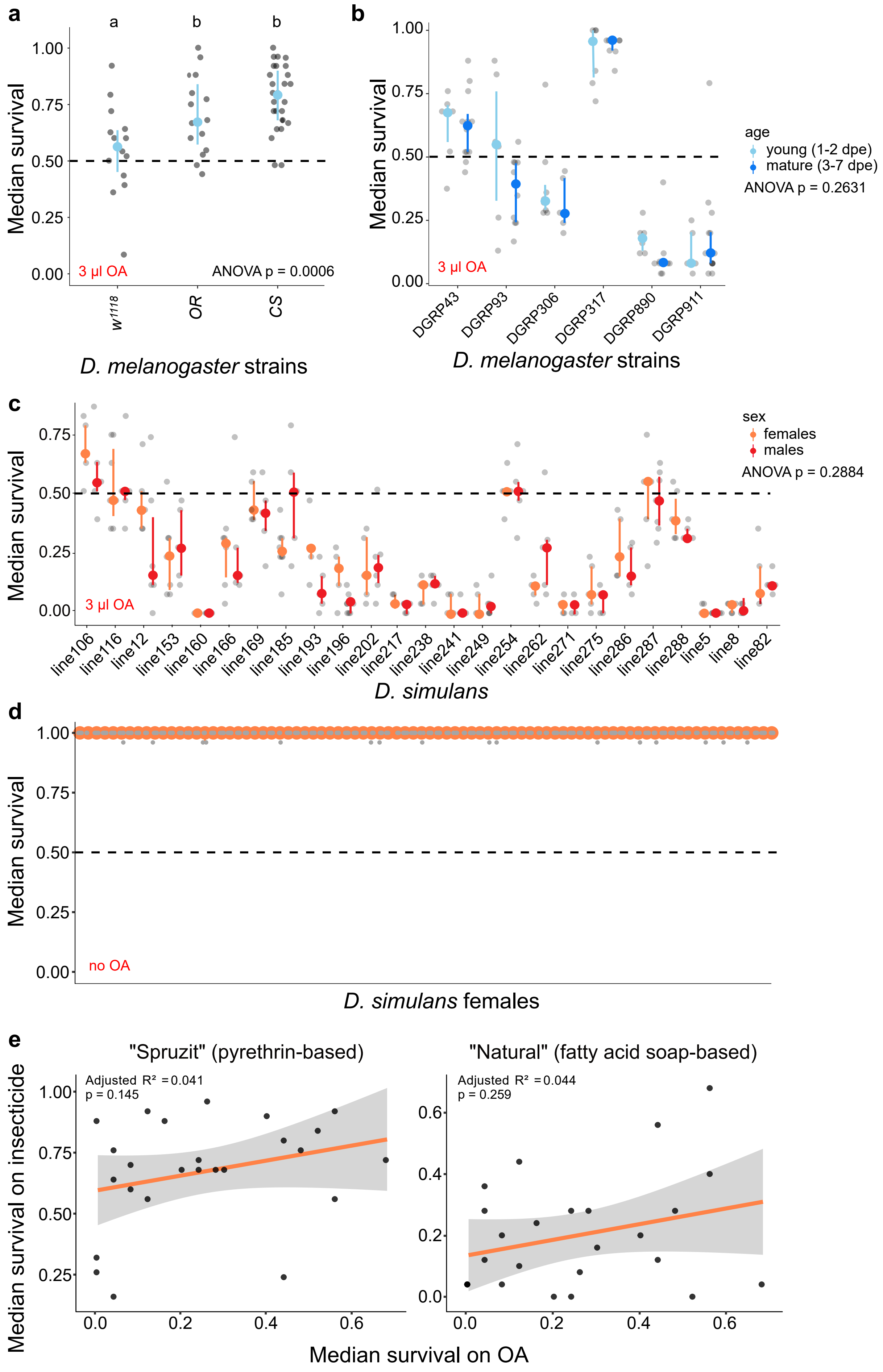

Figure S1. Additional phenotypic characterization of OA resistance in *D. melanogaster* and *D. simulans*.
(a) OA resistance of common *D. melanogaster* laboratory strains (w^{1118}, Oregon-R and Canton-S) for males and females combined.
(b) OA resistance of a subset of DGRP strains in young (1-2 days post-eclosion, dpe) and mature (3-7 dpe) mixed sex flies.
(c) OA resistance of males and females of a subset of *D. simulans* strains.
(d) Median survival of *D. simulans* strains (along the x-axis) in the absence of OA.
For (a-d) Colored dots represent median values, colored whiskers indicate interquartile ranges, and grey dots correspond to individual replicates. Statistical differences were assessed using ANOVA followed by Tukey's post hoc test. Raw data are available in File S1.
(e) OA/insecticide (containing either pyrethrin or a mixture of medium chain fatty acids) median survival correlations for a subset of *D. simulans* strains. Raw data are available in File S2.
