# Supplementary material for: Genome-wide association studies identify new candidate genes and tissues underlying resistance to a natural toxin in drosophilids
Source: G3 (Bethesda). 2026 Feb 16;16(4):jkag032. doi: 10.1093/g3journal/jkag032 (PMC13042304; doi:10.1093/g3journal/jkag032)
Supplement: jkag032_Supplementary_Data [file jkag032_supplementary_data.zip › Figure_S2_G3-2026-406569.pdf]

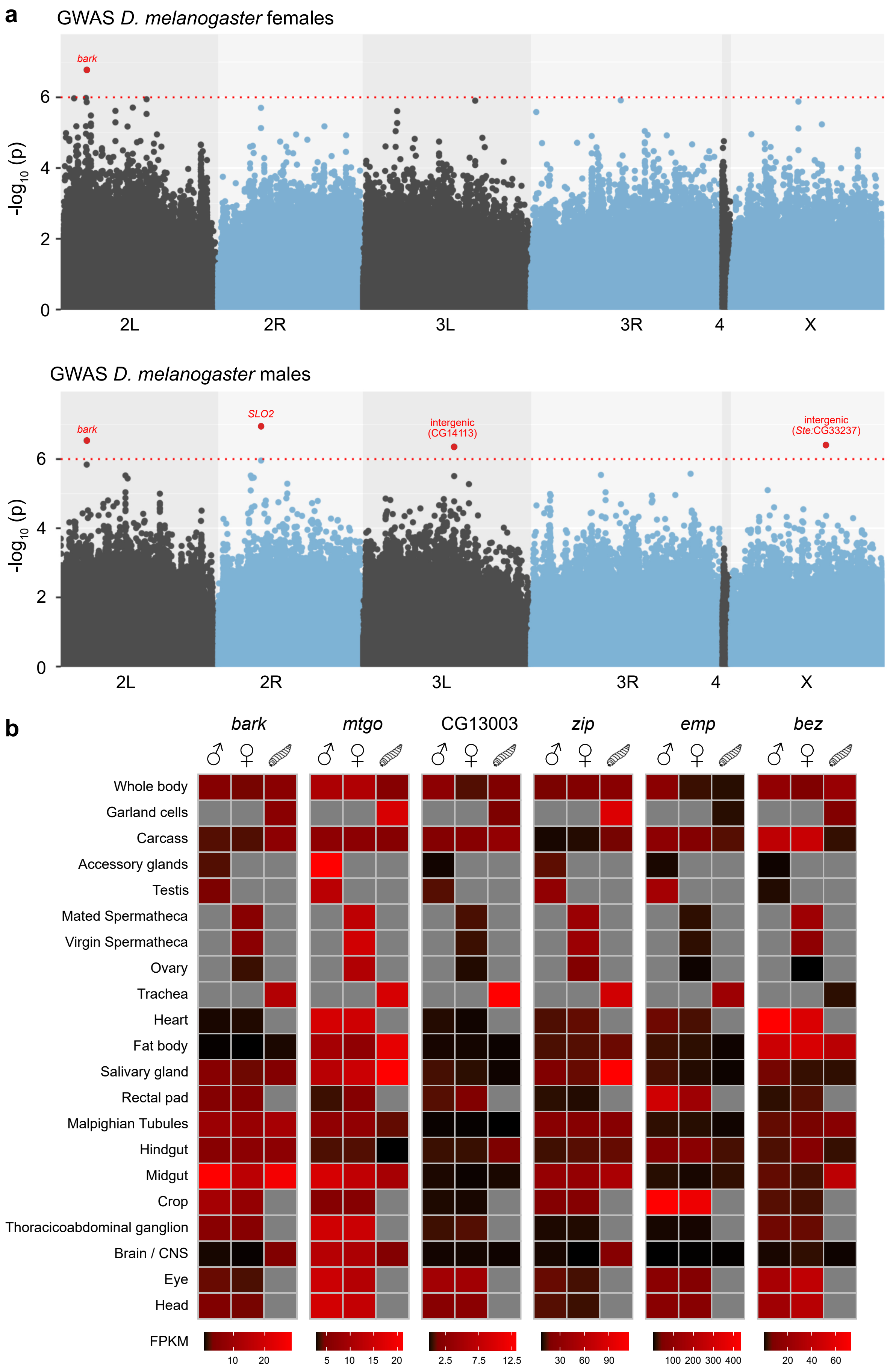


**Figure S2. GWAS of OA resistance in female and male *D. melanogaster*, and tissue-specific expression of candidate OA resistance genes.**
(a) Manhattan plot of the GWAS of OA resistance in *D. melanogaster* (DGRP), based on median survival of females and males. Each dot represents a SNP, plotted on the x-axis according to its genomic position. The y-axis shows $-\log_{10}(p)$ values for the association between genotype and OA resistance. The red dotted line marks a conservative arbitrary threshold at $p \leq 1\times10^{-6}$. SNPs exceeding this threshold are highlighted in red. For intergenic SNPs, the closest gene is indicated. Raw data are available in File S3.
(b) Heatmap showing expression profiles of the candidate genes across tissues, based on FlyAtlas 2 data. Relative expression levels are depicted on a black-to-red scale. Grey indicates no data are available.
